# Policy implications of medical tourism development in destination countries: revisiting and revising an existing framework by examining the case of Jamaica

**DOI:** 10.1186/s12992-015-0113-0

**Published:** 2015-07-04

**Authors:** Rory Johnston, Valorie A. Crooks, Meghann Ormond

**Affiliations:** Department of Geography, Simon Fraser University, 8888 University Drive, V5A 1S6 Burnaby, BC Canada; Cultural Geography, Environmental Sciences, Wageningen University, Wageningen, NL The Netherlands

**Keywords:** Jamaica, Medical tourism, Health services, Health policy, Qualitative methods, International medical travel, Trade in health services, Caribbean

## Abstract

**Background:**

Medical tourism is now targeted by many hospitals and governments worldwide for further growth and investment. Southeast Asia provides what is perhaps the best documented example of medical tourism development and promotion on a regional scale, but interest in the practice is growing in locations where it is not yet established. Numerous governments and private hospitals in the Caribbean have recently identified medical tourism as a priority for economic development. We explore here the projects, activities, and outlooks surrounding medical tourism and their anticipated economic and health sector policy implications in the Caribbean country of Jamaica. Specifically, we apply Pocock and Phua’s previously-published conceptual framework of policy implications raised by medical tourism to explore its relevance in this new context and to identify additional considerations raised by the Jamaican context.

**Methods:**

Employing case study methodology, we conducted six weeks of qualitative fieldwork in Jamaica between October 2012 and July 2013. Semi-structured interviews with health, tourism, and trade sector stakeholders, on-site visits to health and tourism infrastructure, and reflexive journaling were all used to collect a comprehensive dataset of how medical tourism in Jamaica is being developed. Our analytic strategy involved organizing our data within Pocock and Phua’s framework to identify overlapping and divergent issues.

**Results:**

Many of the issues identified in Pocock and Phua’s policy implications framework are echoed in the planning and development of medical tourism in Jamaica. However, a number of additional implications, such as the involvement of international development agencies in facilitating interest in the sector, cyclical mobility of international health human resources, and the significance of health insurance portability in driving the growth of international hospital accreditation, arise from this new context and further enrich the original framework.

**Conclusions:**

The framework developed by Pocock and Phua is a flexible common reference point with which to document issues raised by medical tourism in established and emerging destinations. However, the framework’s design does not lend itself to explaining how the underlying health system factors it identifies work to facilitate medical tourism’s development or how the specific impacts of the practice are likely to unfold.

## Background

As international trade in services has become increasingly desirable, health services have been identified as a promising export sector by many national governments and business consultancies worldwide [1, 2]. Medical tourism is one form of health services export that has recently attracted considerable attention, often reported as a sector that is quickly growing and immensely valuable [3, 4]. Most generally, medical tourism describes the temporary movement of a patient outside the health system of their habitual country of residence for the purpose of purchasing medical care [5]. Here we consider medical tourism to be restricted to patients who *intentionally* travel to another country primarily for medical care and pay for their care privately. We impose this restriction in order to exclude ‘cross-border’ patients who are referred abroad by their home health systems and thereby face a different profile of health and financial risks than do medical tourists acting on their own [6–8].

Southeast Asia provides what is perhaps the best documented regional example of medical tourism development and promotion, with numerous hospitals and national and provincial governments strategically targeting the sector for investment and further growth in the wake of the 1998 Asian Financial Crisis. Private hospitals in Singapore, Thailand, and Malaysia are all known to be attracting significant numbers of medical tourists from both within and outside the region [9, 10]. These countries’ national governments have been very supportive of medical tourism, creating policies and organizations to increase the export of medical services. The policies and strategies adopted among these countries commonly include the creation of visas specifically for medical tourists [11, 12], the reduction or elimination of taxation on imported medical equipment and supplies [13, 14], incentives and/or requirements for international hospital accreditation [13], and international marketing efforts that advertise the high quality of medical care available [13, 15]. When taken together, these initiatives demonstrate a regional concentration of similar promotional and development initiatives among proximate health care markets competing for international privately-paying patients.

The Caribbean provides another example of region-wide interest in medical tourism. While Cuba has long supported its health system by treating medical tourists, its early pursuit of the sector was initiated by the collapse of economic support from the Soviet Union [13]. St. Lucia, Barbados, the Cayman Islands, and Jamaica are among the countries in the Caribbean whose governments and hospitals have more recently identified medical tourism as a strategic priority for economic development [16–19]. These latter countries and their health care sectors share similarities in their common development of facilitative frameworks for private health sector investment, their formation of national committees to guide the development of policies that support medical tourism, their participation in international trade conferences promoting medical tourism, and/or their development of new facilities whose primary purpose is to treat medical tourists [16, 17, 19]. These activities are an indication of the sustained and widespread interest across many Caribbean countries in developing their health services sectors for export. However, little is known about the perspectives, information, and goals driving these planning initiatives in the Caribbean or how a medical tourism sector is understood to interact with the existing health systems of these countries. This article provides a case study of the planning and development activities for the medical tourism sector in Jamaica using primary qualitative data collected in on-site fieldwork. This account provides insights into how and why the sector is emerging there and the implications its development for health policies.

### Jamaican context

In the past five years, Jamaica has undertaken all of the medical tourism development initiatives common to Caribbean nations described above [20–24]. A small-island state with a population of 2.7 million, Jamaica is classified as an upper-middle-income country, although the country is deeply indebted and its economy is characterized by long-term stagnancy [25, 26]. Recreational tourism is one of the country’s largest economic sectors, representing roughly 8 % of direct contribution to the country’s gross domestic product in 2013 (26 % of the total including indirect and induced effects) and 7 % of direct employment [27, 28]. The tourism industry – highly enclavic, dependent on imports, and with significant levels of foreign investment – is concentrated on the western and northern coasts, with Kingston, the capital and country’s economic centre, located on the south-central coast [29]. Medical tourism has been framed by local proponents as a viable and lucrative sector that should be developed as a natural extension of the country’s existing tourism product [30–32].

In spite of the country’s economic difficulties, Jamaica has a relatively robust, if severely under-resourced, health system. Jamaica’s training programs for both physicians and nurses are internationally respected, the country has a strong foundation of primary care delivery that has secured low rates of communicable disease and infant mortality, and the country possesses an established network of secondary and tertiary care centres in urban centres [33]. This strong provider network and Jamaica’s small size have resulted in geographically accessible health care throughout the country [34]. Jamaican citizens have universal access to publicly funded primary care clinics and secondary and tertiary hospitals without user fees, although most Jamaicans with private health insurance or the means to afford out-of-pocket payments prefer to access primary and secondary care at private facilities due to negative perceptions of the public system [33, 35, 36]. This is a source of inequitable access between poorer and wealthier Jamaicans, with a greater range of care options and resources available to the portion of the population with a lower burden of poor health because of their higher socio-economic status [37]. Overall, the vast majority of inpatient services are provided in public facilities while the private sector provides roughly half of the total share of outpatient services [38].

While there are few financial barriers facing Jamaicans seeking public medical care, the country’s weak economy has proved a major challenge in sustaining adequate financing for the country’s medical facilities across both the public and private health sectors [33, 35]. Funding shortfalls have contributed to major staffing shortages among nurses and specialists nationwide, especially as they face relatively few barriers migrating to the more lucrative health systems of Canada, the United Kingdom, and the United States [39, 40]. These health system factors serve as important context for Jamaica’s developing medical tourism industry, which is focused on the country’s private health care sector. Jamaica has eight private hospitals [33], although there is little third party data available on their quality, range of services, or utilization. Reports on Jamaica’s overall health sector capacity do not provide detailed breakdowns of the public/private share of the country’s 4,800 hospital beds or its health human resources, nor are there reliable collations of information about the private health sector in general [41].

### Study rationale and goal

In this article we seek to contribute to the ongoing scholarly conversation about the impacts of medical tourism on health systems, their workers, and their local users (*e.g.* [10, 42–44]) by documenting and critically assessing the planning and perceptions surrounding the development of medical tourism in the Caribbean country of Jamaica. We do this by applying Pocock and Phua’s [45] conceptual framework of the policy implications for destination countries posed by the development of medical tourism. Pocock and Phua previously developed this framework, published in *Globalization & Health,* following a retrospective review of the literature on the well-established medical tourism sectors in Malaysia, Singapore, and Thailand. Their purpose in developing this conceptual framework was not to assess the systemic elements of ‘successful’ health services exporters, but instead to highlight and organize common health policy implications raised by medical tourism for destination countries. Table 1 outlines the health system impacts and their associated policy implications identified by the framework, which are structured around core health system elements into the domains of governance, financing, delivery, regulation, and health human resources.

**Table 1:** Pocock and Phua’s (2011) conceptual framework for medical tourism’s policy implications

	Policy Implications
Governance	• number and content of health sector commitments in multi- and bilateral trade agreements
(Legislation and Planning)	• regional trade blocs promoting trade in health services
	• national medical tourism committees or agencies
	• creation of medical tourism travel visas
Financing	• increase in out of pocket payments
(Fundraising and Payment)	• increasing interest in internationally portable health insurance
Delivery	• growth of private health sector
(Service Provision and Infrastructure)	• foreign direct investment in health infrastructure
Regulation	• public and private sector quality control
(Protocol Creation and Enforcement)	• international accreditation of health facilities (*e.g.* Joint Commission International)
	• number of medical tourist visits facilitated by brokers
Human Health Resources	• distribution of specialists between public and private health sector
(Training and Supply of Care Personnel)	• future human resource capacity (re: training, availability,professional to population ratios)

The broad scope of the health system domains and the high-level considerations identified in Pocock and Phua’s framework encourage its application beyond the original context where it was developed. We use the existing framework to organize our findings from fieldwork in Jamaica that seeks to understand how medical tourism is being developed there. As Jamaica is *prospectively* developing a medical tourism sector located outside of the region where the conceptual framework was derived, we extend the framework and its implications outside its original geographic and temporal scope while also providing the first in-depth account of Jamaica’s plans for medical tourism. This approach explores the wider relevance of the framework and its implications in a new context and identifies numerous additional system impacts and policy implications from the Jamaican experience that refine or are not captured by the existing Pocock and Phua framework. These include new policy implications in every existing domain of the existing framework and the creation of an entirely new domain, that of ‘consumers’. Taken together, we believe our revised framework, derived from new insights gleaned from Jamaica, provides a more comprehensive range of relevant policy implications emerging from the development of medical tourism for monitoring and policy development worldwide.

## Methods

Building on our existing Caribbean fieldwork [16, 42, 46], Jamaica was identified as a highly relevant site to prospectively explore how medical tourism is being pursued by Caribbean governments and health care providers that do not have an existing reputation for health services export. Ongoing monitoring of Caribbean media and online medical tourism promotion outlets such as the Medical Tourism Association and the International Medical Travel Journal indicated Jamaica’s interest in developing a medical tourism sector. This was further reinforced by the presence of Jamaican representatives at the 2011 Global Healthcare Congress where the first two authors were in attendance. These initial impressions of Jamaica’s promotional activities for medical tourism made it clear that the country does not have sizeable existing inflows of international patients but that its government and care providers are working to build a medical tourism sector. This indicated that the country would serve as a suitable case to explore how medical tourism is emerging in a new location using Pocock and Phua’s framework to identify the health policy implications of its development.

After receiving ethics approval from Simon Fraser University’s research ethics board, we travelled to Jamaica in October, 2012, for a two week period of on-site fieldwork. The first author returned to Jamaica for an additional four weeks of fieldwork in June/July, 2013. The research trips were structured within a case study methodological framework and employed a range of qualitative methods to better understand how medical tourism is currently understood and being mobilized in Jamaica. Semi-structured interviews with health system and tourism sector stakeholders; site visits to municipalities, health facilities, and recreational tourism locations; and daily researcher group journaling structured around shared reflective questions were all used to rigorously compile a comprehensive dataset.

### Data collection – semi-structured interviews

While on-site, we sought to interview key-informants that could speak to the planning and development efforts for medical tourism in Jamaica as well as the existing health and/or tourism sectors. Potential interview participants were identified based on reviews of publicly available reports and news coverage of medical tourism in Jamaica, contacting individuals and organizations with roles relevant to medical tourism development, and ongoing referrals from interview participants. We sought to maximize the diversity of key-informants in order to capture a breadth of perspectives. Seventy-six potential participants and organizations were contacted and asked to follow up by phone or e-mail if they were interested in learning more about the study or scheduling a face-to-face interview at a time and location of their convenience. A letter of information outlining the purpose and scope of the study as well as its risks and benefits was provided to participants prior to the interview, whereupon consent was obtained. Whenever possible, interviews were digitally recorded. However, six participants declined to be recorded and requested that only written notes be taken by the interviewer(s).

In total, 18 semi-structured interviews lasting from 30-60 min were conducted with representatives of public and private hospitals, extant and planned; government ministries; organizations charged with overseeing professional and regulatory roles in the medical sector; and individual medical professionals and tourism experts. Interview questions generally inquired about current strengths and challenges within the health system, medical tourism initiatives, and anticipated impacts of a medical tourism sector, though specific questions were tailored in order to speak to each participant’s expertise. Table 2 provides an overview of the participant numbers and their professional domains. Note that the total number is higher than 18 due to some cross-categorization of participants.

**Table 2:** Participant overview

Employment Sector	Number of participants
Governance	7
Academic and Training	4
Private Health Sector	5
Public Health Sector	3
Trade and Development	2

### Data collection – site visits

We based ourselves in Kingston, the Jamaican capital, during both fieldwork periods in order to facilitate interviews and visit Jamaica’s core medical infrastructure. A five-day trip to the country’s recreational tourism hub on the west coast was also conducted to meet with stakeholders and observe tourist and medical facilities there. Site visits were made to five existing medical facilities identified in interviews or news reports as potential locations for medical tourism, as well as two green-field locations positioned as future locations for medical tourism facilities. Site visits involved visiting surrounding communities where the (proposed) facilities are located, touring facilities to view infrastructure and see operations first-hand, and informal conversations with workers and users when appropriate. Detailed written notes were taken by the researchers during and immediately after site visits and were transcribed and uploaded to our secure shared server for later analysis.

At the conclusion of each day during the first fieldwork period, we took one hour to write reflective notes around a set of common questions in order to capture our impressions of the site visits. Following writing, we met for an additional hour to discuss the written observations and interpretations of sites visited and debrief about the details shared in interviews during that day, recording common and divergent issues and interpretations. This daily exercise sought to ensure that a comprehensive range of observations was captured and also to improve interpretative agreement. During the second fieldwork period the first author kept an independent journal that recorded impressions and observations that was later shared among the team.

### Data analysis

Following each fieldwork period, all interview transcripts and field notes were compiled in a secure shared database. The database also holds all reports and news articles that were compiled in preparation for and collected during the research trips. Using a categorization scheme adapted from Pocock and Phua’s framework of policy implications emerging from medical tourism, the plans, issues, and actors referred to across the full dataset were organized into their respective domains (governance, financing, delivery, regulation, health human resources) by the first author. Plans, issues, and actors that did not conform to the existing scheme were noted and categorized separately. Following this, a synthesis of the categorized and non-conforming findings was circulated to the second and third authors to confirm their interpretation and comprehensiveness and achieve consensus. This process resulted in the most consistent of the non-conforming findings being agreed on as our sixth analytical category of ‘consumers’. Our findings from this domain are outlined in the section below following the results that fall within the existing five health policy domains identified by Pocock & Phua’s framework.

## Results

In this section we organize our fieldwork findings according to the health system domains outlined in Pocock and Phua’s framework in order to facilitate comparison and discussion. Their domains are: governance, financing, delivery, regulation, and health human resources. Table 1 (above) provides a summary of how the framework characterized each domain and its associated policy implications. While we acknowledge the clear interrelationships between these five domains, we consider each separately in order to effectively engage with Pocock and Phua’s framework. Each section in our results briefly notes where the implications from the original framework are transferable to Jamaica’s experience, however we focus on the issues and impacts identified from our Jamaican fieldwork that were *not* part of Pocock and Phua’s original policy implications framework. We do so given their prominence in discussions with key-informants and in order to highlight and unpack these new considerations that warrant being factored into dialogues and future studies of the health policy implications raised by medical tourism.

### Governance

With the exception of creating travel visa categories specifically for medical travellers, all of the policy implications resulting from the development of medical tourism outlined by Pocock and Phua’s conceptual framework are also found to be relevant to Jamaica’s sector planning. Our fieldwork revealed two additional impacts relevant to the governance domain not included in their original framework: 1) the expansion of ministerial responsibilities and convergence of inter-ministerial relationships; and 2) the involvement of international development organizations in initiating and sustaining interest in medical tourism. We separately expand on these two impacts below.

Jamaica’s national government has actively supported the development of a medical tourism sector in the country since at least 2007, with political leaders and government ministers from both main political parties expressing their support for specific hospital projects focusing on the international patient market and the broader idea of medical tourism as a viable strategy for economic growth. Most recently, the Ministry of Industry, Investment and Commerce, the Ministry of Tourism, and the Ministry of Health have been jointly involved in laying the groundwork for a medical tourism sector. JAMPRO, an agency of the Ministry of Industry, Investment and Commerce that is responsible for attracting foreign direct investment and promoting Jamaican goods and services for export, has been the most active participant in spearheading sector development. For example, JAMPRO has recruited international consultants to assess the viability of medical tourism and directly participated in trade shows and conferences organized by the United States-based Medical Tourism Association and the regional Caribbean Export Development Agency. It has also convened national stakeholder meetings and drafted an incentive framework to develop policy and encourage private investment in the health services sector. Interview participants made it clear that JAMPRO has played the key leadership role in developing a vision for a Jamaican medical tourism sector and organizing policy development. Participants also indicated that the Ministries of Health and Tourism and the domestic private health sector, while supportive of medical tourism, have participated in a more limited consultative role rather than overseeing policy development or coordinating private investment in health facilities.

Another group of actors involved in medical tourism sector development and related to health sector governance that were raised in the interviews are international development organizations – a group not considered in Pocock and Phua’s framework. In conversations with participants, the Canadian International Development Agency (CIDA) and the Commonwealth Secretariat were both identified as having played some role in advancing the medical tourism sector. These economic development agencies have been directly implicated in the development of medical tourism by helping to fund regional Caribbean conferences on health tourism more broadly and in commissioning and funding the production of strategic reports that explore how to develop medical tourism in Jamaica. A participant further noted that funding provided by such agencies earmarked for economic development might be used in the future to commission additional studies or to offset tax incentives for growth in the medical tourism sector, demonstrating the potential scope and policy relevance of this consideration.

### Financing

Our fieldwork found limited consideration of medical tourism’s likely influences on patterns of care financing. If discussed at all, out-of-pocket payments were understood to be the likely source of health care financing for any near-term plans for medical tourism. However, in light of Jamaica’s proximity to the United States and interest in attracting American patients and insurers, intersections between international hospital accreditation and international health insurance portability not identified in the original Pocock and Phua framework were regularly raised by participants from the medical sector. Progress on international health insurance portability was reported to be undermined by the lack of local medical facilities with international hospital accreditation. This was compounded by the significant financial barriers to successfully obtaining international accreditation facing medical facilities. As such, while accessing foreign insurance funds was perceived by most participants to be a long-term goal, out-of-pocket payments are understood to be the most likely financing method for international patients seeking care in the immediate future.

### Delivery

Jamaica’s experience in planning for medical tourism thus far broadly confirms the applicability of Pocock and Phua’s anticipated policy-relevant issues in the delivery domain. Our fieldwork revealed three new implications relevant to this domain: 1) the utilization of existing, underutilized private sector supply; 2) an anticipated increase in for-profit health care delivery; and 3) the creation of ‘offshore’ medical facilities with deep ties to neighbouring health systems and foreign investment. These impacts are described below.

Our fieldwork found two distinct strategic models for the initial development of the Jamaican medical tourism sector being pursued simultaneously. In the first, medical tourism will primarily serve as an additional market for the existing stock of private hospitals. Jamaica has a number of private hospitals located in urban districts with nearby international airports. Participants reported that these existing private hospitals are both locally well-regarded as reputable and having under-utilized capacity. This excess capacity was identified by participants as being well suited to meeting the demands of the international patient market because of the range and quality of services available. However, it was acknowledged that there are few international patients currently accessing these facilities outside of the Jamaican diaspora. International hospital accreditation was consistently identified as the largest barrier to immediately accessing the international market. This is because accreditation processes and the costs of the renovations they typically demand pose a large and immediate financial barrier.

A second project identified by participants as being spurred by the prospect of medical tourism involves the construction of a private wing within Cornwall Regional Hospital, an existing public hospital in Montego Bay. Planning for this new wing intends for it to be closely integrated into the infrastructure and operations of the existing public hospital, but for it to be privately financed and managed. Although it was noted by one participant that the primary reason for creating the wing is to both improve the quality of care available to tourists who injure themselves while visiting Montego Bay and to bring in medical tourists, they were careful to explain that its services would be available to private paying Jamaicans as well. It was also emphasized that the additional funds generated by private services would directly cross-subsidize a new public children’s hospital affiliated with Cornwall Regional Hospital, without which the new facility could not sustainably operate.

In the second model, new hospitals would be built primarily to serve (medical) tourists. This is evidenced in two recent projects. In the first project, American Global MD, a group of American physicians headed by a Jamaican-American, proposes an entirely new 75-bed private hospital explicitly framed as a medical tourism destination financed with foreign investment and built in Montego Bay, the largest city on the west coast. This facility would be staffed by American physicians, while the local population would fill nursing and ancillary positions. The second project is spearheaded by a charitable organization in the resort town of Negril, also on the west coast. The Negril International Hospital plans to serve as the hub of a larger retirement community that will target foreign and diasporic buyers. Its staffing model mirrors American Global MD’s approach, with foreign physicians supplying the specialty labour and medical tourists comprising the bulk of their cases. Local patients would be served in a charitable capacity with medical tourists subsidizing their cost of care. This specific example of seasonal and year-round retirement migration supporting a medical tourism facility was favoured among numerous participants who see the realization of Jamaica’s existing plans to attract foreign and diasporic retirees to the country contingent on improving the quality of local medical care. Medical tourism is seen as providing the additional revenues required to viably sustain such health system improvements.

### Regulation

Our fieldwork found that the issue of international hospital accreditation previously identified by Pocock and Phua’s framework was by far the most prominent regulatory issue raised in conversations with Jamaican stakeholders. Participants who had engaged with international consultants or workshops/conferences on medical tourism reported having the importance of hospital accreditation in internationally marketing hospitals emphasized to them. However, international hospital accreditation was widely perceived to be a large and costly barrier to attracting international patients, and concerns were shared that the accreditation might be clinically redundant to local standards already in place. This perception prompted some participants to raise the possibility of intra-regional coordination of health care standards and regulation as they relate to medical tourism, an issue not considered in Pocock and Phua’s framework. These same participants usually raised the example of harmonized health and safety standards developed for the spa and wellness tourism sector by the Caribbean Spa and Wellness Association as a model of how intra-regional coordination of the medical tourism sector at the pan-Caribbean scale could occur. With organizational assistance from CARICOM and funding from international development organizations, the Caribbean Spa and Wellness Association organized a committee to identify best practices from existing spa standards in North America, Europe, and Asia in order to address an existing regulatory gap. The result of this extensive review process was the creation of up-to-date health and safety regulations for spa and wellness service facilities shared across Caribbean countries. This case was presented as an example of successful regional regulation within the wider health tourism sector that could be copied in developing a regional hospital accreditation standard.

### Health human resources

Discussions with Jamaican stakeholders and reviews of policy and media documents revealed that all health human resources-related policy implications identified by Pocock and Phua’s framework are relevant to Jamaica’s plans for an expanded medical tourism sector. We identified three additional policy-relevant issues pertaining to the health human resources domain: 1) health worker training as a marketing tool; 2) the increased overall demand for health human resources; and 3) the increasing international mobility and altered circulation of health human resources.

Jamaica’s health workers were repeatedly referred to by participants as the country’s greatest asset in developing the country’s medical tourism sector. This confidence stemmed from the long-standing international ties underpinning local training practices and standards that have produced high quality and culturally familiar medical traditions comparable to other Commonwealth countries. This confidence was also informed by the long history of Jamaican diaspora working as physicians and nurses in the health systems of Canada, the United Kingdom, and the United States. It was thought that the established familiarity of Jamaican health workers in these target medical tourist markets and their reputable and interpretable training backgrounds would serve as a powerful marketing tool for health service exports from Jamaica.

Participants were largely unconcerned by the potential for medical tourism to reduce the availability of physicians in the public health system due to increased demand. This perception was not the same across the two different models of medical tourism being discussed (*i.e.*, domestic services export versus temporarily importing foreign health workers). With regard to the first model where Jamaican health care facilities and health workers provide the services, participants consistently reported that the existing arrangement between Jamaican private and public health care provides evidence that the two streams of delivery are not in competition, as most specialists participate in care delivery across both systems and nursing wages are comparable between them. Meanwhile, in the second model, facilities plan to recruit foreign physicians to work in Jamaica on a temporary basis at offshore hospitals focusing on medical tourists. This was seen as a way for foreign physicians and their families to go on vacation, to generate additional income or, as in the case of the Negril International Hospital, to provide charitable care for locals as well. Thus, this second model was perceived as having limited potential to impact the existing Jamaican health system because of these facilities outsourcing much of their own specialist labour.

Given the extreme nursing shortage in Jamaica induced by extraordinary out-migration, participants generally gave deep consideration to the potential impacts on nursing availability in the public system resulting from additional demand for private health care, regardless of staffing formats for physicians. Nurse migration from Jamaica was chiefly presented as being motivated by a desire to earn more income abroad. Medical tourism was generally understood to be a counter-weight that would provide higher wages domestically, incentivizing nurses to remain in Jamaica and ultimately increasing the local availability of health workers. This widely shared outlook was contrasted by a minority who cautioned that the higher wages that could be supported by private care provision for international patients could serve to attract the most qualified or senior care providers from the public sector. Altogether, participants demonstrated greater sensitivity to medical tourism’s potential to impact the local availability of nurses than physicians.

### Consumers

The domain of ‘consumers’ was not identified in Pocock and Phua’s original framework. Our fieldwork in Jamaica, however, raised a number of critical policy-relevant questions about how perceptions of the international patient market are compelling policy action and, in turn, how a larger volume of health service exports might alter the composition of health system users in medical destinations. Participants demonstrated a consistent vision of the composition of the international patient market and, consequently, the patients that Jamaican stakeholders are working to attract. There was a widespread perception that Jamaica will tap into a large market of under- or un-insured patients from the United States. While Canada and the United Kingdom were sometimes mentioned as additional markets, the United States is the predominant target market shaping stakeholders’ current expectations and planning efforts.

Participants typically did not identify patients from within the Latin American and Caribbean region or the Jamaican diaspora as potential medical tourists informing current planning. Once prompted, participants generally expressed enthusiasm for attracting patients from wherever they might come, but it was clear that medical tourists are commonly interpreted as being patients from high-income settings who are not emigrants with ties to the country. This clarifies who are understood to be medical tourists among sector stakeholders in Jamaica and therefore which international patients are being most actively sought and planned for.

## Discussion

The policy implications raised by the planning for medical tourism in Jamaica and their significance are discussed below within the domains of Pocock and Phua’s existing framework. Table 3 provides a synthesis of our additional key considerations.

**Table 3:** Additional policy implications from medical tourism development

	Original Policy Implications	Additional Implications Identified from Jamaican Case
Governance	• number and content of health sector commitments in multi- and bilateral trade agreements	• expanding/conflicting ministerial responsibilities and novel inter-ministerial relationships
(Legislation and Planning)	• regional trade blocs promoting	• involvement of international
	• trade in health services	• development organizations and foreign for-profit industry organizations in developing medical tourism sectors
	• national medical tourism committees or agencies	
	• creation of medical tourism travel visas	
Financing	• increase in out of pocket payments	• intersections between international hospital accreditation and international health insurance portability
(Fundraising and Payment)	• increasing interest in internationally portable health insurance	
Delivery	• growth of private health sector	• utilization of existing private sector oversupply
(Service Provision and Infrastructure)	• foreign direct investment in health infrastructure	• increased for-profit healthcare delivery
		• cross-subsidization schemes to explicitly benefit locals
		• development of enclavic medical tourism facilities
Regulation	• public and private sector quality control	• regional development and coordination of healthcare standards
(Protocol Creation and Enforcement)	• international accreditation of health facilities (*e.g.* Joint Commission International)	
	• number of medical tourist visits facilitated by brokers	
Human Health Resources	• distribution of specialists between public and private health sector	• health worker training as marketing tool
(Training and Supply of Care Personnel)	• future human resource capacity (re: training, availability, professional to population ratios)	• increasing international mobility and circulation of healthcare labour (including importation)
		• increased demand for different types of health human resources with varying supply
Consumers		• narrow conceptions of international patient market and inflated projections informing sector development
(Composition and Number of Patients)		
		• increased utilization of health services by emigrant diaspora

### Governance

In a departure from Pocock and Phua’s original framework, the involvement of Jamaica’s Ministry of Tourism in planning for medical tourism sector development reinforces that, regardless of discursive debates around medical tourism versus medical travel (*e.g.* [47, 48]), the frame being used to develop policy for the sector in emerging destinations hinges on existing experience with recreational tourism. This approach is producing novel intersections between government ministries, evident in the involvement of both the Ministry of Health and Ministry of Tourism in the consultation and policy planning being led by Jamaica’s foreign investment and export promotion corporation, JAMPRO. These intersections may result in incoherent or competing priorities informing the development of the sector. This was suggested by the regular conflation among many stakeholders that were interviewed between their enthusiasm and plans for a ‘health tourism’ product – including wellness retreats, complementary and alternative treatments – with the narrower, biomedical focus of ‘medical tourism’. The Ministries involved and their respective roles demonstrate that medical tourism is being pursued primarily as an economic, not health system, development project in Jamaica. While the Ministry of Health’s support for the sector may result in outcomes that benefit and integrate with the local health system, its role in developing and regulating an emergent medical tourism sector could also serve as a distraction from its core mandate of overseeing local health (care) concerns.

The role of international aid and development agencies as well as professional medical tourism industry associations observed in the Jamaican context was also not previously captured in Pocock and Phua’s framework. We think their role most coherently fits within the domain of ‘governance’ given the role that the various groups and institutions have played in informing and supporting sector development. Because of the potential for medical tourism to negatively impact the equitable delivery of health care [43], the support of international development organizations for the medical tourism sector raises important questions for both their accountability and overall coherence of different initiatives’ goals. Any efforts by development organizations to promote medical tourism as an economic development strategy must work to ensure that health equity is not compromised. This could be through concurrent efforts to advance initiatives such as universalizing health insurance coverage and transparent healthcare cross-subsidization schemes in jurisdictions that are encouraged to develop medical tourism.

### Financing

Medical tourism’s anticipated impacts on health care financing in Jamaica are consistent with those highlighted by Pocock and Phua’s framework, but our results indicate financing impacts are likely to be staged according to phases of sector development. While out of pocket payments are perceived to be a matter of necessity, access to an increasingly portable American and European health insurance market was described as a key long term goal. The original framework does not capture the relationship between access to international insurance markets and international hospital accreditation that was identified by Jamaican interview participants. This widely shared perception helps to further explain the growing popularity of international hospital accreditation. Instead of only being framed as a market response to individual patient demands for recognizable seals of quality, international hospital accreditation also positions hospitals as viable participants in an emerging, internationally portable insurance environment [10]. International accreditation may be critical in anticipating current and future flows of insured international patients. Should the volume of international medical travel grow significantly, it will make it increasingly important to have a clear understanding of the strengths and limitations of the various international hospital accreditation standards that are being used to underwrite the risks of international care.

### Delivery

The numerous new private hospital projects being planned for in Jamaica that are reliant on foreign patients confirm the original framework’s general insight that increasing interest in medical tourism is accompanied by the growth of the private health sector. Our findings further demonstrate that medical tourism is also spurring significant *qualitative* changes to health services organization and delivery. For example, medical tourism’s direct role in informing the planning for a new private wing in a public hospital with the expectation that it will cross-subsidize public access to care is not captured by the broad original consideration of a growing private health sector. Similarly, in systems such as Jamaica where participants reported excess capacity in the private health care sector (a key element in narratives explaining the shift towards the international market among Asian hospitals (*e.g.* [13]), turning to international patients in order to meet existing capacity can be interpreted as more efficient utilization of existing private resources rather than overall growth of the private sector. This alternate interpretation of how the private health sector might engage in or respond to medical tourism raises questions about how under-utilized private hospital beds inform interest in medical tourism among hospitals and governments.

In their plans to focus their services on international patients, the proposed ‘offshore’ hospitals in Jamaica invert the existing model of medical tourism as a niche market for established hospitals. These plans are echoed in other Caribbean projects such as Health City in the Cayman Islands and American World Clinics in Barbados. These projects raise important health equity concerns for the health systems that host them [16–19]. In primarily employing foreign specialists and treating foreign patients, these facilities would present an especially severe form of two-tiered care, particularly if associated with the enclavic communities being planned for foreign retirees. The focus on increasing foreign investment in the health sector that these offshore hospitals represent is likely to be amplified by Jamaica’s weak economic position. Interview participants from the existing private health sector (dominated by a mixture of not-for-profit hospitals and small, for-profit, physician-owned speciality clinics) clearly articulated concerns that they would face difficulty accessing the financial resources necessary to upgrade their facilities to the quality capable of attracting international patients, suggesting immediate future development may be driven by foreign investment.

This above contrast in local versus foreign financial capacity raises further questions regarding the scale and structure of foreign investment in health services relative to domestic ownership, not just an anticipated absolute increase. Given Jamaica’s already crowded private hospital market, how will new, foreign-owned private hospitals impact existing locally-owned ones, especially with their attendant for-profit structure? Medical tourism development strategies that focus on inviting new, foreign investment could ultimately diminish domestic ownership in the private healthcare market over time and consolidate foreign ownership of health services. Contextual dynamics such as these demonstrate that while ‘increased foreign direct investment in health services’ is a fruitful point of departure for considering the impacts of medical tourism on a healthcare environment, providing even a rough outline of the existing private healthcare landscape prior to the development of medical tourism is critical to unpacking how such an outcome might actually unfold on the ground.

### Regulation

As highlighted by Pocock and Phua’s model, international hospital accreditation is closely tied to Jamaica’s plans to develop a medical tourism sector. Stakeholder interviews demonstrated that established medical tourism destinations’ experiences with international accreditation are informing Jamaica’s policy development for the sector. International consultants and organizations promoting medical tourism have driven home the importance of obtaining international hospital accreditation to Jamaican medical tourism stakeholders by raising successful examples from Malaysia, Thailand, and Costa Rica. This suggests that international hospital accreditation’s growth may have, in part, achieved self-reinforcing momentum as it becomes a core ingredient in the standard development formula being disseminated by international consultants promoting medical tourism. This requires investigation to determine if its high costs, relative to the economic conditions of the destinations pursuing them, are justified by a clinically relevant improvement in service quality. The Caribbean Spa and Wellness Association’s success in creating their own set of therapeutic standards demonstrates that cooperation in regulating emerging economic sectors related to health and wellness most broadly is a viable route for Caribbean countries. Should medical tourism development follow suit and serve to initiate the development of a regional hospital accreditation scheme, the sector could ultimately standardize and improve the quality of hospital care in emerging destinations without incurring the costs of more expensive international accreditation regimes. This potential is unlikely to be realized, however, should marketing considerations and international insurance portability provide the dominant reasons for seeking accreditation.

### Human resources

The original Pocock and Phua framework focuses on medical tourism’s influence on specialist behaviour, particularly the potential for a larger private patient market to further incentivize specialists to reduce their participation in the public healthcare sector. This impact was largely interpreted as a non-issue by the majority of interview participants, being explained in three interrelated ways. Firstly, the largest medical tourism projects being pursued in Jamaica plan to rely on foreign specialist labour, insulating the local health system from losing its own supply of workers. Secondly, the kinds of procedures envisioned as constituting the bulk of medical tourist cases were elective, particularly plastic surgery and orthopaedic care, and thereby thought to insulate critical care specialities from negative impacts. Lastly, there was reported to be a great degree of existing participation across the public and private systems among specialists. It was reported that specialists seek out cases (and thereby payment) wherever available and rarely opt to solely practise in the private sector.

While the paucity of information on public versus private sector participation by medical specialists makes it impossible to independently confirm these perceptions above, they illustrate the outlooks driving the acceptability of medical tourism in emerging destinations. If medical tourism is understood to primarily deal in elective treatments, the kinds of specialist labour in increased private demand are not perceived as a threat to health equity. Arguably, this dominant outlook understates potential health equity impacts by neglecting to engage with the inevitable linkages between facilities, specialties, and overall health resources that occur in real-world health systems. Participants’ heightened sensitivity to the potential for medical tourism to place stress on the insufficient local supply of nurses demonstrates the relevance of these linkages. Their concerns also highlight the need to differentiate the impacts of medical tourism on different health professions. Whereas Pocock and Phua’s original framework focuses on medical tourism’s impacts on specialists and largely ignores other groups of health workers, future research examining medical tourism’s impacts should seek to understand how it influences nurses, specialists, and generalists as they vary both in supply and in the degree of control they have over where and how often they work.

Lastly, we propose the addition of ‘health worker training as a marketing tool’ to the ‘human health resources’ domain because of both how often participants emphasized the quality and familiarity of Jamaican health workers as a justification for developing medical tourism sector and given the discussion of training elsewhere in the literature as an asset to sector development (*e.g.*, [49, 50]). This consideration is significant in understanding the factors that initiate and sustain interest in developing medical tourism and a key factor driving the perceived viability of the sector in Jamaica. Furthermore, formal and informal connections between health systems, particularly international patterns of health worker training and employment, are potentially powerful predictive precursors of international patient flows.

### Consumers

Our fieldwork demonstrates that the beliefs surrounding who medical tourists are and the scope of their numbers are critical in understanding the enthusiasm among countries seeking to become medical tourism destinations. While there is no doubt that there are American patients willing to travel for medical care, the widespread uptake among health and tourism sector stakeholders in Jamaica of inflated projections that have been disseminated by industry promoters and consultants such as the Medical Tourism Association and Deloitte’s 2008 report on medical tourism [51] is alarming. These sources’ wide-ranging estimates and implicit expectation that the American health system will continue to fail in adequately insuring its own population have been critiqued as overly narrow and inaccurate elsewhere (*e.g.* [3, 52]). Regardless, articulating who sector stakeholders are targeting when developing medical tourism is important in unpacking the likely economic returns and health system impacts that the sector will produce. For example, recent work from Lunt *et al.* [53] and other scholars examining medical tourism (*e.g.* [52, 54]) suggest that the international patient market is most accurately understood as relatively small and reliant on existing social ties between particular ‘source’ and ‘destination’ countries, rather than a large, unattached market of atomised patient-consumers with a willingness to travel anywhere for the care they want. While less economically tantalizing, broadening the popular conception of medical tourists beyond the image of the uninsured American patient or wait-listed Canadian or Briton to focus on regional and diasporic populations could promote the development of health services exports that are likely to have greater uptake while also being more relevant and accessible to the domestic population.

### Study limitations

As researchers with no personal or cultural ties to Jamaica, the issues that were raised in interviews and the manner in which they were framed by participants may be different than those produced by a local researcher. Controversial issues or factors that might negatively impact foreign perception of Jamaica (*e.g.*, high crime and violence rates) may have been omitted by participants, even unintentionally. As our findings emerged from a qualitative case study design, the policy implications raised here are not generalizable to all jurisdictions developing medical tourism sectors. This is not a true limitation, though, as qualitative research seeks to achieve transferability and not generalizability. Further to this, the considerations identified here and in the original framework are intended to raise implications for further investigation rather than serve as a prescriptive account.

## Conclusions

This analysis demonstrates that the domains of Pocock and Phua’s framework for identifying policy-relevant implications of the medical tourism sector provide a useful common reference point for exploring the development of medical tourism in new contexts. The original framework’s inclusion of very specific issues (*e.g.* ‘creation of medical tourism visas’) alongside broad ones (*e.g.* ‘growth of private health sector’) and the absence of distinction between the drivers and impacts of medical tourism provides great latitude in adapting it to different locations and stages of development, including Caribbean countries like Jamaica. While its basic design does not supply explanatory power or trace the relationships between the pre-existing conditions that facilitate medical tourism and the impacts of the practice, the Pocock and Phua framework does provide a useful organizational scheme to identify and compile additional policy-relevant observations. For example, most of the additional policy implications that we identified in the Jamaican context are logical extensions of the original five domains in the framework. Our addition of ‘consumers’ to the framework as a new policy-relevant domain expands Pocock and Phua’s original conceptualization to capture issues emerging from our work in the Jamaican context. It is likely that the numerous previously unidentified policy implications and new domain reported in the current analysis hold true for the wider Caribbean and are also relevant beyond the region.

We believe that future health services research examining medical tourism can further and meaningfully contribute to refining the Pocock and Phua framework by identifying additional implications emerging from the development and operation of the sector in other countries and regions beyond those already considered. Such research will assist in assessing the global transferability of the framework. Future studies can also advance our understanding of the policy implications of medical tourism development by undertaking comparative analyses of the implications specific to particular framework domains, which will help us to understand the role that local context plays in shaping how they are occurring in particular ways in specific places. At the same time, such research will further our understanding of common trends that are structuring the development of medical tourism worldwide and the scope of its health system impacts and policy implications despite such differences.

## References

[1] Lautier M (2008). Export of health services from developing countries: the case of Tunisia. Soc Sci Med.

[2] Smith RD, Chanda R, Tangcharoensathien V (2009). Trade in health-related services. Lancet.

[3] Connell J (2013). Tourism Manage.

[4] Horowitz MD, Rosensweig JA, Jones CA (2007). Medical tourism: globalization of the healthcare marketplace. Med Gen Med.

[5] Ormond M. Medical tourism. In The Wiley-Blackwell Companion to Tourism. 2^nd^ edition. Edited by Lew AA, Hall CM, Williams A. Malden, MA: John Wiley & Sons; 2014:425-434.

[6] Glinos IA, Baeten R, Maarse H (2012). Purchasing health services abroad: Practices of cross border contracting and patient mobility in six European countries’. Health Policy.

[7] Lunt N, Carrera P (2010). Medical tourism: assessing the evidence on treatment abroad. Maturitas.

[8] Penney K, Snyder J, Crooks VA, Johnston R. Risk communication and informed consent in the medical tourism industry: A thematic content analysis of Canadian broker websites. BMC Med Ethics. 2011;12:17. 10.1186/1472-6939-12-17 PMC3189886 21943392

[9] Heung VCS, Kucukusta D, Song H (2010). A conceptual model of medical tourism: implications for future research. J Travel Tourism Marketing.

[10] Whittaker A (2008). Pleasure and pain: medical travel in Asia. Global Pub Health.

[11] International Medical Travel Journal. New visa exemptions to attract more medical tourists to Thailand. Int Med Travel J 2013. [http://www.imtj.com/news/?entryid82=413889]

[12] Yuen M. Malaysia’s visa change is a boost for healthcare tourism. eTurbo News (2009) at <http://www.eturbonews.com/13394/malaysias-visa-change-boost-healthcare-tourism>

[13] Chee HL (2010). Medical tourism and the state in Malaysia and Singapore. Global Soc Policy.

[14] Turner L (2007). ‘First world health care at third world prices’: globalization, bioethics and medical tourism. Bio Societies.

[15] Ormond M (2013). Neoliberal Governance and International Medical Travel in Malaysia.

[16] Adams K, Snyder J, Crooks V, Hoffman L (2014). Medical tourism in the Caribbean: a call for cooperation. WIMJ Open.

[17] Connell J (2013). Medical tourism in the Caribbean islands: a cure for economies in crisis?. Island Stud J.

[18] Feinsilver JM (2010). Fifty years of Cuba’s medical diplomacy: From idealism to pragmatism. Cuban Studies.

[19] de Arellano AB R. Medical tourism in the Caribbean. Signs. 2011;36:289–96. 10.1086/655908 21114073

[20] Jamaica Observer. Boost for Ja's medical tourism plan. Jamaica Observer. September 25, 2010. [http://www.jamaicaobserver.com/news/Boost-for-Jas-medical-tourism-plan_7994302]

[21] Benzler D. The 4th Annual WMT&GHC Announces Jamaica Promotions Corporation (JAMPRO) as an Exhibitor. Med Tourism Mag | Blog 2011. [http://www.medicaltourismmag.com/blog/2011/10/the-4th-annual-wmtghc-announces-jamaica-promotions-corporation-jampro-as-an-exhibitor/]

[22] International Medical Travel Journal: JAMAICA: New report on medical tourism in Jamaica. Int Med Travel J 2011. [http://www.imtj.com/news/?EntryId82=291763]

[23] Braham A. PM Encourages Investment in Medical Tourism. Jamaica Information Service 2014. [http://jis.gov.jm/pm-encourages-investment-medical-tourism/]

[24] Jamaica Promotions Corporation. Annual Report 2012/2013. Kingston Jamaica Promotions Corporation; 2013. [http://www.jamaicatradeandinvest.org/sites/default/files/annual_reports/JAMPRO-AnnualReport2012_2013.pdf]

[25] Tennant D (2011). Factors impacting on whether and how businesses respond to early warning signs of financial and economic turmoil: Jamaican firms in the global crisis. J Econ Business.

[26] World Bank: Jamaica. Jamaica Data 2013. [http://data.worldbank.org/country/Jamaica]

[27] World Travel and Tourism Council. Travel & Tourism Economic Impact 2014: Jamaica. London; 2014. [http://www.wttc.org/~/media/files/reports/economic%20impact%20research/country%20reports/jamaica2014.ashx]

[28] Holzner M (2008). Tourism and economic development: The beach disease?. Tour Manage.

[29] Williams DA, Deslandes D (2008). Motivation for service sector foreign direct investments in emerging economies: insights from the tourism industry in Jamaica. Round Table: Commonwealth J Int Affairs.

[30] Brown I. Jamaica going for a piece of medical tourism market. Jamaica Observer 2011. [http://www.jamaicaobserver.com/news/Jamaica-going-for-piece-of-medical-tourism-market_8821139]

[31] Cunningham A. Jamaica Must Focus More on Health Tourism - Mascoll. Jamaica Gleaner 2013. [http://jamaica-gleaner.com/gleaner/20130617/lead/lead2.html]

[32] Jamaica Information Service: Jamaica to Benefit From New Venture in Health Tourism. Jamaica Information Service 2007. [http://jis.gov.jm/jamaica-to-benefit-from-new-venture-in-health-tourism/]

[33] Plummer JM, Roberts PO, Leake PA, Mitchell DI (2011). Surgical care in Jamaica in the Laparoendoscopic Era: challenges and future prospects for Developing Nations. Perm J.

[34] Pan American Health Organization. Jamaica. Regional Outlook and Country Profiles. Washington, DC; 2012 [http://www.paho.org/hq/index.php?option=com_docman&task=doc_view&gid=25344&Itemid=]

[35] Scott E, Theodore K (2013). Measuring and explaining health and health care inequalities in Jamaica, 2004 and 2007. Rev Panam Salud Públ.

[36] Bourne PA (2010). Self-reported health and medical care-seeking behaviour of uninsured Jamaicans. N Am J Med Sci.

[37] Scott E, Theodore K (2013). Measuring and explaining health and health care inequalities in Jamaica, 2004 and 2007. Rev Panam Salud Publica.

[38] Anderson-Jackson L, McGrowder DA, Bourne PA, Crawford T, Whittaker WHA (2009). Response of patients to the introduction of a private magnetic resonance imaging service in Western Jamaica. N Am J Med Sci.

[39] Cawich S (2013). Leadership in surgery for public sector hospitals in Jamaica: strategies for the operating room. Perm J.

[40] Salmon ME, Yan J, Hewitt H, Guisinger V (2007). Managed migration: the Caribbean approach to addressing nursing services capacity. Health Serv Res.

[41] Tomblin Murphy G, MacKenzie A, Guy-Walker J, Walker C (2014). Needs-based human resources for health planning in Jamaica: using simulation modelling to inform policy options for pharmacists in the public sector. Hum Resour Health.

[42] Johnston R, Crooks VA (2013). Medical tourism in the Caribbean region: a call to consider environmental health equity. W Indian Med J.

[43] Hopkins L, Labonté R, Runnels V, Packer C (2010). Medical tourism today: What is the state of existing knowledge?. J Public Health Policy.

[44] Lautier M (2014). International trade of health services: Global trends and local impact. Health Policy.

[45] Pocock NS, Phua KH. Medical tourism and policy implications for health systems: a conceptual framework from a comparative study of Thailand, Singapore and Malaysia. Globalization Health. 2011;7:12. 10.1186/1744-8603-7-12 PMC3114730 21539751

[46] Johnston R, Crooks VA, Snyder J, Whitmore R (2015). ‘‘The major forces that need to back medical tourism were…in alignment’’: ‘championing development of barbados’ medical tourism sector. Int J Health Serv, Serv.

[47] Kangas B (2011). Complicating common ideas about medical tourism: gender, class, and globality in yemenis’ international medical travel. Signs.

[48] Turner L (2013). Transnational medical travel. Camb Q Healthc Ethic.

[49] Ormond M, Thomas F, Gideon J (2013). Harnessing “diasporic” medical mobilities. I. Migration, Health and Inequality.

[50] Walton-Roberts M. International migration of health professionals and the marketization and privatization of health education in India: from push-pull to global political economy. Soc Sci Med, in press. 10.1016/j.socscimed.2014.10.004 25445935

[51] Deloitte Center for Health Solutions. Medical Tourism: Consumers in Search of Value. 2008. Washington, DC. [https://www.academia.edu/9144718/Medical_Tourism_Consumers_in_Search_of_Value_Produced_by_the_Deloitte_Center_for_Health_Solutions]

[52] Ormond M, Sulianti D (2014). More than medical tourism: lessons from Indonesia and Malaysia on South–South intra-regional medical travel. Current Issues Tourism.

[53] Lunt N, Jin KN, Horsfall DG, Hanefeld J (2014). Insights on medical tourism: markets as networks and the role of strong ties. Korean J Soc Sci.

[54] Noree T, Hanefeld J, Smith R. UK medical tourists in Thailand: they are not who you think they are. Globalization Health. 2014;10:29. 10.1186/1744-8603-10-29 PMC4038702 24885204

